# Interpreting global variations in the toll of COVID-19: The case for context and nuance in hypothesis generation and testing

**DOI:** 10.3389/fpubh.2022.1010011

**Published:** 2022-10-19

**Authors:** Roger M. Stein, David L. Katz

**Affiliations:** ^1^NYU Center for Data Science and Stern School of Business, New York University, New York, NY, United States; ^2^True Health Initiative, Hamden, CT, United States

**Keywords:** COVID, pandemic, health policy, statistical methods, obesity, machine learning, health economics, lifestyle factors

## Abstract

**Key points:**

As of January 2022, the COVID-19 pandemic was on-going, affecting populations worldwide. The potential risks of the Omicron variant (and future variants) still remain an area of active investigation. Thus, the ultimate human toll of SARS-CoV-2, and, by extension, the variations in that toll among diverse populations, remain unresolved. Nonetheless, an extensive literature on causal factors in the observed patterns of COVID-19 morbidity and cause-specific mortality has emerged—particularly at the aggregate level of analysis. This article explores potential pitfalls in the attribution of COVID outcomes to specific factors in isolation by examining a diverse set of potential factors and their interactions.

**Methods:**

We sourced published data to establish a global database of COVID-19 outcomes for 68 countries and augmented these with an array of potential explanatory covariates from a diverse set of sources. We sought population-level aggregate factors from both health- and (traditionally) non-health domains, including: (a) Population biomarkers (b) Demographics and infrastructure (c) Socioeconomics (d) Policy responses at the country-level. We analyzed these data using (OLS) regression and more flexible non-parametric methods such as recursive partitioning, that are useful in examining both potential joint factor contributions to variations in pandemic outcomes, and the identification of possible interactions among covariates across these domains.

**Results:**

Using the national obesity rates of 68 countries as an illustrative predictor covariate of COVID-19 outcomes, we observed marked inconsistencies in apparent outcomes by population. Importantly, we also documented important variations in outcomes, based on interactions of health factors with covariates in other domains that are traditionally not related to biomarkers. Finally, our results suggest that single-factor explanations of population-level COVID-19 outcomes (e.g., obesity vs. cause-specific mortality) appear to be confounded substantially by other factors.

**Conclusions/implications:**

Our methods and findings suggest that a full understanding of the toll of the COVID-19 pandemic, as would be central to preparing for similar future events, requires analysis within and among diverse variable domains, and within and among diverse populations. While this may seem apparent, the bulk of the recent literature on the pandemic has focused on one or a few of these drivers in isolation. Hypothesis generation and testing related to pandemic outcomes will benefit from accommodating the nuance of covariate interactions, in an epidemiologic context. Finally, our results add to the literature on the ecological fallacy: the attempt to infer individual drivers and outcomes from the study of population-level aggregates.

## Motivation and background

The trajectory of the COVID-19 pandemic is still evolving, and is unlikely to be fully understood and elaborated until history and hindsight confer clarity and render their verdicts—which may take many years. Even so, the *great variation* in reported COVID-19 cause-specific mortality rates^1^ around the world has precipitated a vast and still burgeoning literature that posits a variety of “likely” causes for such variation. Some of these proposed associations may yet prove obsolete when the full country-specific human costs of the pandemic are updated and enumerated. It is the nature of this work, that efforts to examine cause and effect at this juncture are “snapshots,” fixed in time, even as the target—the ultimate toll of the pandemic in lives lost and harmed—remains in motion.

However, some salient associations^2^ have been suggested [e.g., (1)]. Among these, there appears to be a consistent, strong, and biologically plausible link between obesity and adverse COVID-19 outcomes [e.g., (2)]. The bulk of the COVID-19 cause-specific mortality toll in the United States appears to be associated with the broader, and related matter of poor cardiometabolic health and to issues of health inequality [e.g., (3)]. A number of authors have asserted similar associations, often taken as attributions, with respect to a number of countries and regions [e.g., (4, 5)].

While direct *associations* between certain biological factors and COVID-19 outcomes may provide a comfortable explanation for variation in pandemic outcomes, the matter appears to be far more nuanced normatively.

**Example 1: Obesity vs. COVID-19 cause-specific mortality**
The implications of obesity for health seem to be both conditional and non-linear. In affluent countries, individual obesity tends to be associated with relative indigence, limited nutritional options, economic insecurity, and resource scarcity. In contrast, in relatively indigent countries, individual obesity is often associated with affluence, food security, economic security and reduced physical labor; and resource access [e.g., (6)].

The simple interaction in the Example between the two factors, one biological and one socioeconomic, is only an illustrative case of a much broader set of analytic challenges. It demonstrates a simple example of one of a much larger collection of subtle statistical associations on which our collective knowledge of the impact of public health policies and interventions rests.

As a result, in many cases, coarse initial findings that provide simple explanations (and sound-bites) for the relationship between lifestyle factors and COVID-19 outcomes, may become strained, or at least less compelling, when examined more fulsomely in the context of additional real-world factors.

In the data we examined, which includes a sampling of measures and metrics from a variety of fields both within and outside of epidemiology, it was not uncommon for several of factors of interest to exert influences on, and also be influenced by, one or more of the others. Policy responses, for example, may reflect many different phenomena, from political inclinations to simple resource availability to the timing of the spread of the pandemic to more remote regions. Resource availability may in turn suggest variations in access to acute medical care. And so on, since variations in baseline health, variations in nutriture and food security, and many other factors are often intertwined and self-reinforcing.

We argue that understanding the “causal” factors that drive variable COVID-19 cause-specific mortality and morbidity counts around the world (however these are measured and wherever those tallies conclude), requires careful consideration of not just one or two prominent predictor covariates (such as obesity or hypertension) in isolation, but rather the analysis of constellations of covariates and interactions both within the health domain and from (nominally) exogenous sources such as economics, public policy and civil engineering.

In this article, we offer a preliminary perspective on such pandemic research. Our objective is to demonstrate, through examples, that a “view from altitude” that considers an inventory of domain specific covariates, and employs both traditional statistical methods and more flexible machine learning techniques, can provide a much richer perspective on COVID-19 outcomes that is also less prone to error. In contrast, it can highlight that the data may not be sufficient to support an hypothesis or assertion. Our results suggest caution in interpreting research that suggests simple, univariate or linear relationships among the variables studied and COVID-19 outcomes. We provide examples of a number of potential concerns in drawing conclusions from cursory analyses of pandemic data, and explore the implications for achieving a more reliable and nuanced understanding of cause and effect.

Importantly, in this article, we do not posit any associations between *specific* covariates and population outcomes. Rather, we present several stylized results, based on real data, to demonstrate the *challenges* in doing just that, particularly when the data is observed at the population level.

## Methods

### Data

In total, we compiled data on 68 nations spanning a range of geographic regions. We drew on disparate sources (what we termed *base datasets*) to compile the data used in this analysis.^3^ In all cases, the data was collected from public sources. All of these sources reported data at the domicile (country) level.

To form a common data set (the *composite data set*), we integrated data from these different sources, many of which used bespoke naming conventions, terminology, binning units of analysis, and aggregation methodologies over different, sometimes overlapping, time periods. In addition, because some of the data items (e.g., the policy response of a particular country at a particular time) were textual in nature, we developed a number of internal conventions for mapping text descriptions to consistent discrete labels. We also created mappings between analogous categories in different data sets (e.g., for covariates tied to age groups, where age groups might be defined differently).

The base data sets fell loosely into six broad categories:

- Epidemiological outcomes;
- Health and lifestyle behaviors and markers;
- Measures of national-level economic activity;
- Demographic information relating to each country's population composition;
- Proxies for the robustness of each country's national infrastructure; and
- Indicators of national policy responses to COVID-19 along a number of dimensions (travel, gatherings, etc.).

The final composite data set included data on: COVID-19 case- and cause-specific mortality-rates; population-level health statistics (obesity, hypertension, etc.); national economic indicators (unemployment, median income, etc.); demographic information including population density (age cohorts, etc.); proxies for national- and health-infrastructure and connectivity (hospital beds per capita, life expectancy, international travel, etc.).

We augmented these with information on national policy measures, which we coded to create consistent labels. These additional fields included coded data on the s*peed and stringency* of various nationa*l pandemic response policies*, including those relating to: COVID-19 testing; contact tracing; travel restrictions; social gathering restrictions; workplace closure mandates; school closure mandates; and shelter in place/stay home orders.

Details on the sources and conventions we used in assembling our dataset can be found in Appendix D of the online supplemental information.^4^

## Statistical analysis

The primary analytical tools we applied in this research were common statistical routines (i.e., OLS, hypothesis tests, etc.); and basic so-called “machine learning” tools [i.e., CART, a prototypical recursive partitioning algorithm, described in (18)]. These tools are readily available in both open source and commercial software packages. We performed our analysis using the R platform (19) and the rpart package (20).

It is notable that in each of their domains, OLS and CART are among the most widely known and widely used algorithms for estimating multivariate models from empirical data. One reason for this is that they are also representative of the simplest approaches in their respective classes.

Our objective was not to build the best predictive models, or for that matter, to build even good predictive models, but rather to try to describe relationships among the various candidate factors in our study.

Alternative models and covariates would likely yield different results than those we show, and more sophisticated analytical approaches would similarly produce different results. Some of the effects we report could well be diminished, though others might be amplified. But the two methods we have selected are often used “out of the box” in a large proportion of the published literature on the causes of COVID-19 health outcomes. More specifically, the marginal value of more sophisticated model forms is unclear, given the issues in data that we describe.^5^

We do not describe either approach in detail, though most readers will be familiar with OLS [a detailed treatment may be found in (22, 23); or other standard texts].

However, some readers may not be as familiar with CART, a class of statistical estimation techniques called *recursive partitioning* algorithms. Briefly, this type of algorithm produces a tree structure that has been optimized (based on a given objective function) to ensure that each conditional split in the tree results in the maximum amount of differentiation between the original data and the two subsets resulting from splitting the original data. A tree structure is obtained by recursively splitting the data, starting with the full data set, and then splitting each resulting subset, and then splitting each of those, until some stopping criterion has been met. Those readers seeking additional technical detail on the CART algorithm may find more extensive discussions in e.g., (24–26).

## Results/examples

Since our objective is not to construct a theory of COVID-19 drivers, we will present our results as a series of examples that illustrate various of the key propositions we hope to communicate.

### “First-order” relationships

Because of its prevalence in the published literature to date, in the first portion of this study, we focused on the relationship between *national obesity rates* and *COVID-19 cause-specific mortality rates* for 68 countries, first independently of other covariates, and then jointly with them.

**Figure 1:**
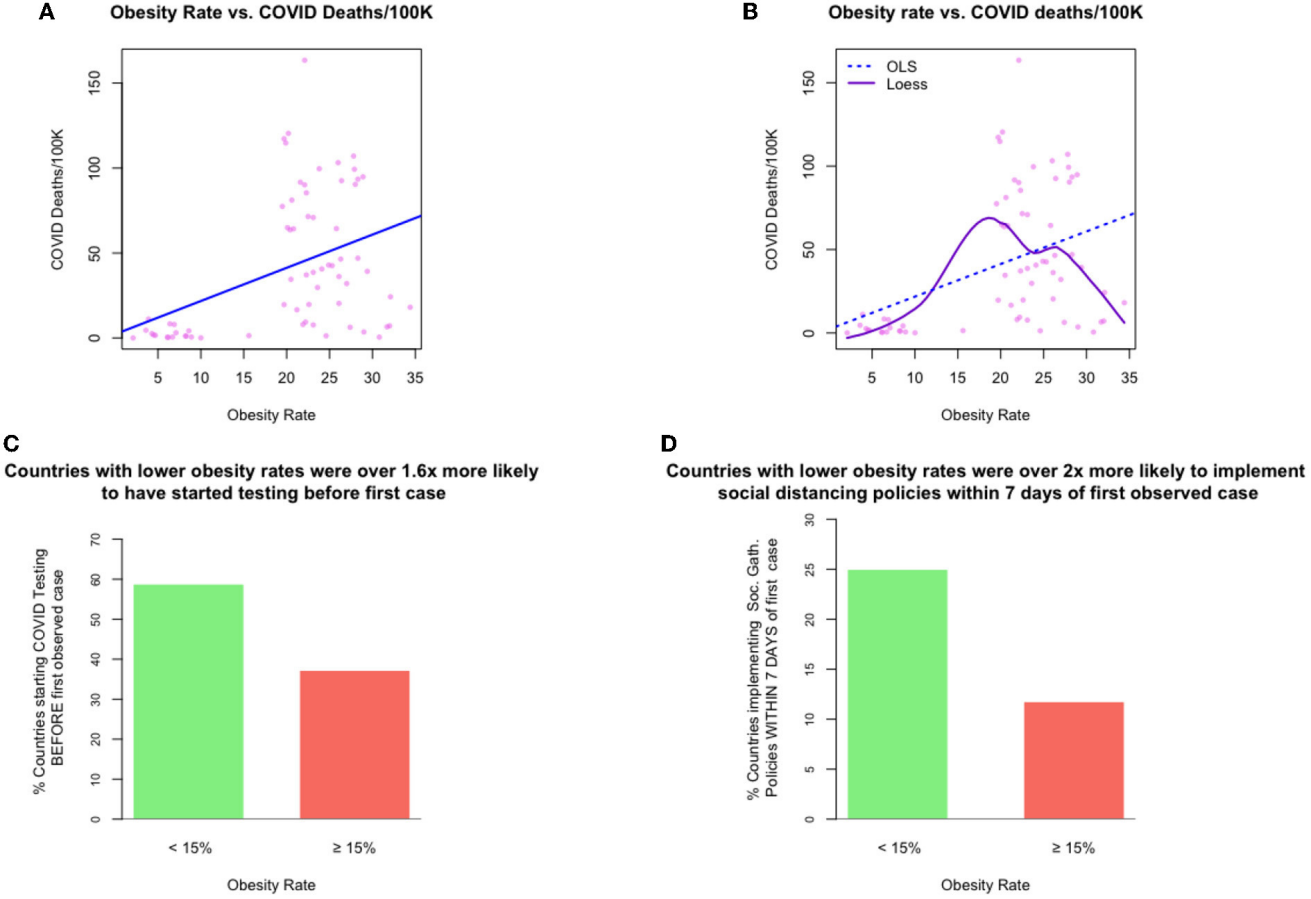
Exhibits for examples 1 and 4: The relationships between obesity rate and other measures of interest. [top left] **(A)** example 1, a (linear) relationship between obesity rate and national-level COVID-19 cause-specific mortality rates, [top right] **(B)** example 2, a (non-linear) relationship between obesity rate and national-level COVID-19 cause-specific mortality rates, [bottom left, right] **(C,D**) example 4, timing of COVID-19 policy response speed for those countries with low vs. obesity.

**Example 2: Obesity vs. COVID-19 cause-specific mortality (cont.)**
We explore in more detail the point made in the introduction: that there are often more complex interactions among, and stratifications within, the relationship between obesity rate and other common national-level health measures.
Figure 1A plots national obesity rates against COVID-19 cause-specific mortality rates, for 68 countries. The line line represents a linear mapping, which is the result of estimating an additive model using OLS. From this linear perspective, the relationship looks fairly strong and monotonic.
But note that in the very highest obesity rate regions of the plot (i.e., 30% obesity and higher), all empirical data points are consistently much lower than the expected (predicted) values of COVID-19 deaths, implied by the linear model. Furthermore, the fit of the linear model for cases in which the obesity rate is between 20 and 30% seems to be very far from many of the real observations.
Now consider Figure 1B, where we show a non-linear relationship from a model estimated using *loess* (solid line), a form of non-linear local regression (27), along with the linear model's estimates (dashed line). The loess fit suggests a more nuanced relationship between obesity and COVID-19 deaths. This relationship appears to be neither linear nor monotonic. For low obesity rate countries, those with obesity rates of 15% or lower, the non-linear model agrees directionally with the linear model, suggesting a positive relationship between obesity and cause-specific mortality. However, this model also suggests the *opposite* relationship for high obesity countries (as obesity rises, the associated cause-specific mortality rates *decrease*).^6^

This example highlights the one of the challenges in finding “simple” explanations for the dynamics of COVID-19 cause-specific mortality rates. Furthermore, while COVID-19 outcomes do seemingly vary in tandem with biological markers, such as obesity rate, outcomes also appear to vary, at the population level, with other non-biological factors such as infrastructure; demographics; and policy responses.

**Figure 2:**
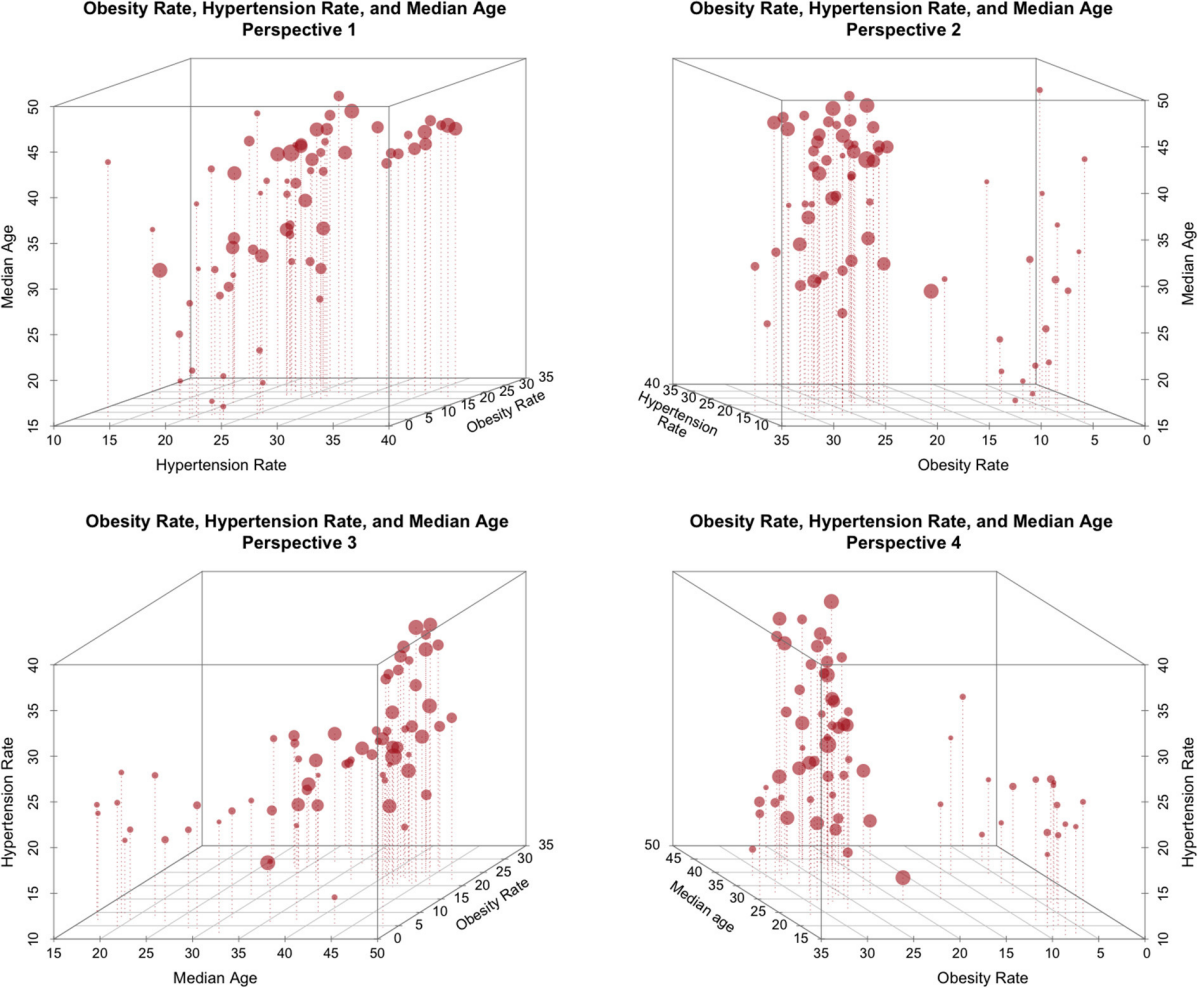
The relationship between obesity rate, Life expectancy, and national hypertension rate (size proportional to COVID-19 deaths/100K).

**Figure 3:**
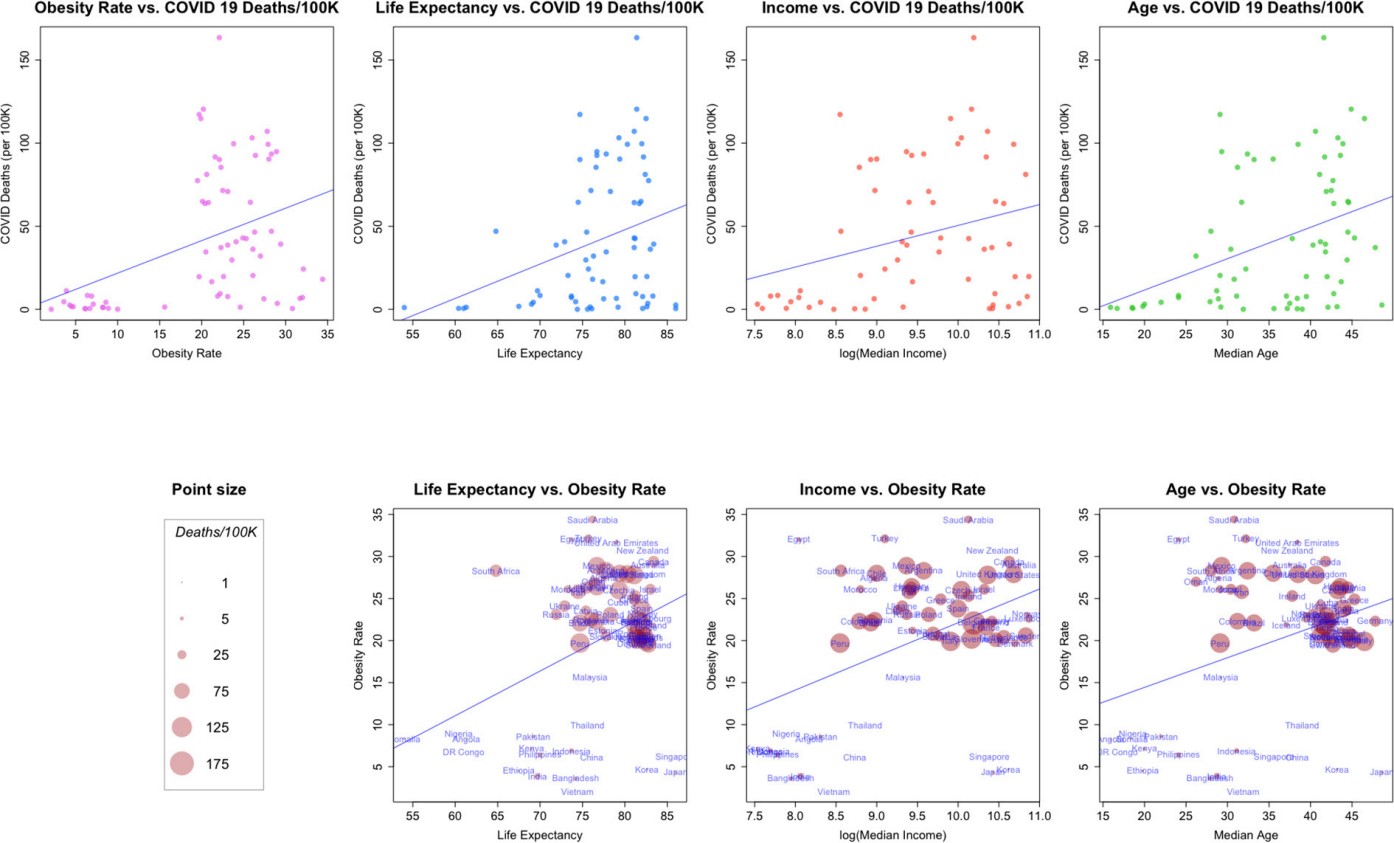
**(Top row)** Relationships between COVID-19 deaths/100K and: Obesity rate, life expectancy, median income and median age. **(Bottom Row)** bivariate relationships, size proportional to COVID-19 deaths/100K.

**Example 3: Interactions among obesity, life-expectancy and hypertension**. Figure 2 shows the joint association, by country, of three factors: obesity rate, median age, and rate of hypertension. The same relationships are shown from four different perspectives. Each point represents one country's data, and the size of each point is proportional to a country's COVID-19 death toll/100K.
From the figure, it is clear that there is substantial structure in the data and that there may be strong relationships among these three factors. Furthermore, there may well be potential causality and/or conditionality effects. However, because the factors shown in Figure 3 are all health-related, a tendency among some authors is to frame these relationships representing a common directional consistency among these coarse measures of overall health, and to thus select the measure most useful for expository purposes, e.g., obesity.

Of course, covariance does not *preclude* causal influence, and might instead indicate a multiplicity of causal pathways, both direct and indirect. There are many reasons, as noted throughout this paper, that obesity is quite plausibly linked to adverse COVID outcomes, which would suggest an ostensibly “direct” path of causality. Obesity is also a risk factor for hypertension (28). To the extent hypertension exacerbates COVID outcomes, and to the extent obesity and hypertension overlap at the population level, the two “risk factors” may each exert *both* independent and *inter-dependent* influences on outcomes. These, in turn, are further complicated by treatment responses, themselves affected by factors of resource availability. Obesity-associated hypertension may either be well controlled, or poorly controlled, depending on to social and economic factors rather than biological ones.

To accommodate this observation, and as the current pandemic has highlighted, the potential for causality/conditionality attribution extends beyond overlapping health markers.

In the next several examples, we extend this approach to also consider markers from other domains. Unsurprisingly, in these settings, COVID-19 outcomes are also associated with differences in policy interventions, economics and infrastructure, and demographics, at the country level.

**Example 4: The relationship between obesity and pandemic policy timing**
Consider Figures 1C,D, which bring into relief the marked differences in policy response timing among those countries with lower obesity rates and those with higher rates. Each bar represents the percentage of countries in the sample that began the respective pandemic intervention policy *before* a COVID-19 case was determined.
This analysis suggests that a country that could plausibly be considered to have a lower obesity rate was 1.6 times more likely to have started COVID-19 testing *before* observing its first COVID-19 case; and about twice as likely to have implemented some form of social distancing policy within 7 days of the first case being observed.
One can imagine a number of reasons for this discrepancy. For example, if lower obesity-rate countries were clustered in geographies far removed from the initial outbreak of the pandemic, these countries may have had more lead-time to devise and plan a response. Or, it might be the case that lower-obesity rate countries are less densely populated, which could curtail transmission; or that those countries have smaller populations, making the rollout of health policy easier; or one or more entirely different explanations.

Example 4 demonstrates a key point: from the data given it is difficult to state convincingly whether (a) COVID-19 death rates are primarily driven by (or even necessarily related to) *either* policy *or* obesity, (b) they are driven by both jointly, or (c) they are driven by some third latent factor. Said differently, if we wish to assert that a country's national-obesity rate is a driver of COVID-19 cause-specific mortality based on a univariate analysis, we should also be willing to accept that countries with low obesity rates were *more* vigilant than those with higher obesity rates, even though the risks to the population is believed to be higher in more obese nations.^7^

We may further pursue this line of inquiry across other domains as well.

**Example 5: Obesity Rate, Life Expectancy, Median Income and Median Age**
In Figure 3, we plot a number of relationships between COVID cause-specific mortality and other factors. The top of Figure 3 shows the relationships between a country's COVID-19 cause-specific mortality rate and several demographic and economic factors, along with a linear model fit to the data.
Taken as a whole, each of the four plots in the top row of the figure suggest a linear relationship between COVID-19 death rates and the second factor, even though the factors are generally from quite different data generating processes (health, health-demographics, and economics). In addition, it appears that the general pattern of the scatterplots is similar across covariates (with the possible exception of that for median income).
The second row of Figure 3 examines combinations of the factors in the first row in a bivariate context, and scales each point so that it's size is proportional to the COVID-19 death rate for a country. Note how the point sizes (COVID-19 cause-specific mortality rates) are often mixed for the same value of a factor. For example, a number of the smallest points (relatively low COVID-19 cause-specific mortality rates) are associated with the highest levels of obesity. These plots suggest again that in general, each of the factors may be associated, but perhaps not linearly.^8^

### Higher-order relationships

Given the many possible interactions and candidate explanatory variables, we found it useful to “scan” for interactions and conditional behavior using tree-based models estimated using CART.

**Figure 4:**
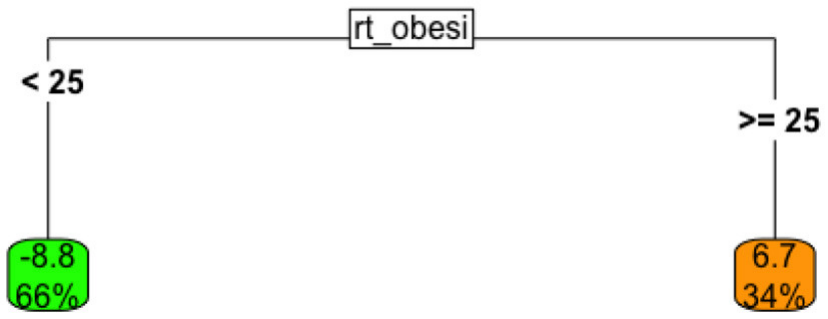
Univariate tree model the relationship between lead-time to the implementation of a national testing policy, given obesity rate.

To fix ideas, Figure 4 shows a very simple tree that provides similar insight into the relationship between policy response *speed* and a country's national obesity rate as was also shown in 2. This model uses the obesity rate directly to estimate the *lead-time* between the start of a specific policy, and the first COVID-19 case identified in that country. Thus, positive numbers imply that the policy was started *after* the first case was identified, while negative numbers imply the policy was started *before* the first case was identified.

This tree suggests that

- When the obesity rate for a country is below 25% (left branch), the country implemented a national testing policy, on average, about 9 days *before* the first local case was observed; this “low-obesity” group represented about 66% of all countries in the data.
- When the obesity rate for a country is > 25% (right branch), the country typically began COVID-19 testing seven days *after* the first case was observed.

Thus, these results conform, in direction, with those observed earlier.

We now move on to more complex examples involving interactions and relationships.

**Figure 5:**
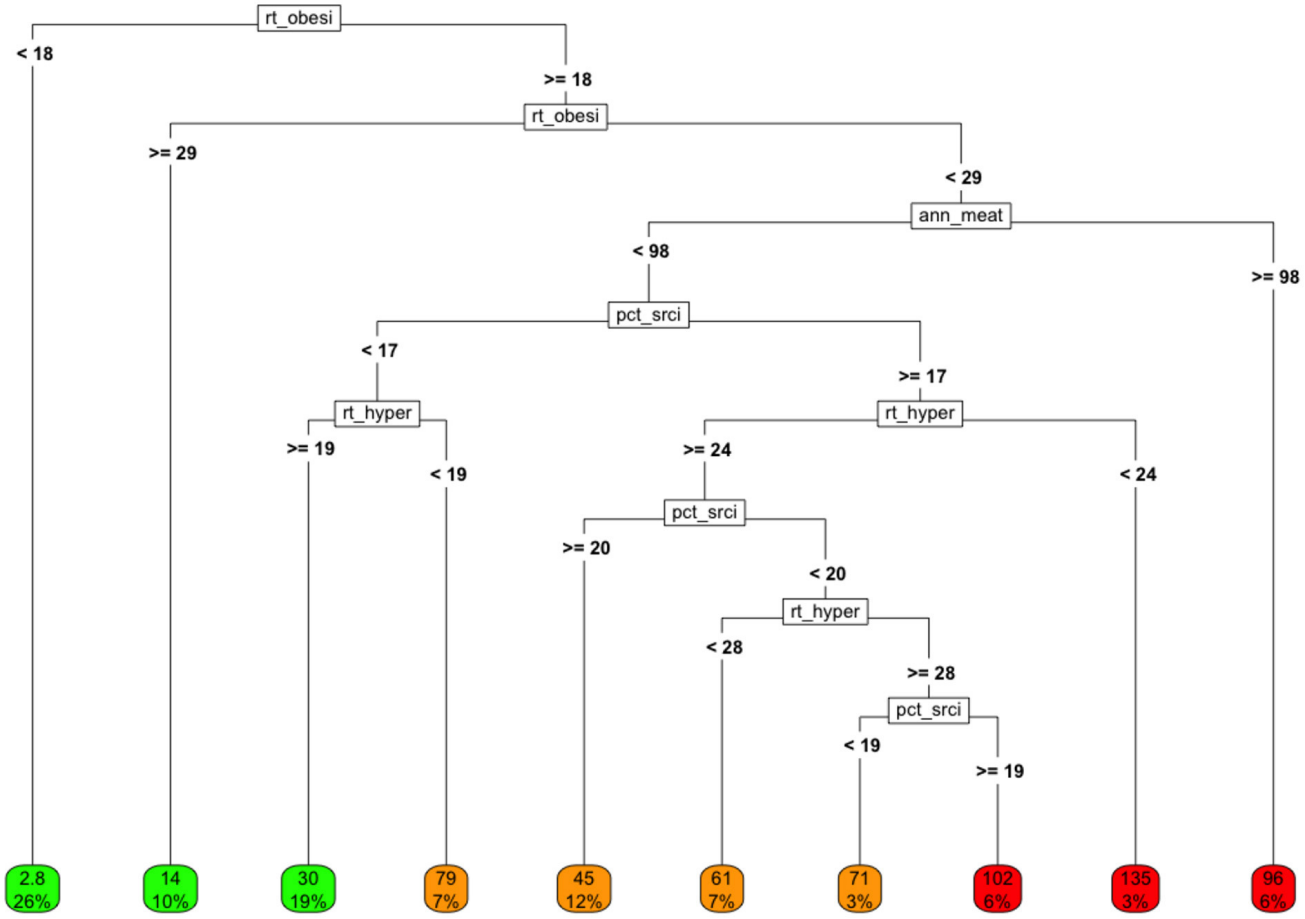
Tree model fit using only health-related markers as covariates and COVID-19 cause-specific mortality (deaths / 100K) by country as the target. Variables in final model: rt_obesity, national obesity rate; ann_meat_consump_kg, average number of kilograms of meat consumed per capita; pct_sr_citz, percentage of population classified as senior citizens; rt_hypertension, percentage of population classified as having hypertension.

**Example 6: COVID-19 Cause-specific mortality and multiple health factors**
We fit a tree-based model using only the covariates in the health marker group to estimate COVID-19 cause-specific mortality, by country. The resulting tree-based model (after pruning) is shown in Figure 5.
The analysis suggests that the *lowest cause-specific mortality* rates occurred in countries with obesity rates that were *either below 18%* or *above 28.9%*. This is consistent with the loess analysis presented earlier.

If we ignore the rightmost node, which represents only a small number of countries, the *highest cause-specific mortality rates* are observed for countries in which:

- The *obesity rate is intermediate* (between 18 and 28.9%)
- The *senior populations tend to be smaller* as a proportion of total population
- The annual per capita *meat consumption is less than about 100kg/person*, and
- The *hypertension rate is relatively high*.

One can imagine exploring whether this clustering might be related to overall low nutritional quality, to the degree of development, etc.

Note that if obesity were truly a linear, isolated (i.e., the *only*) “cause” of adverse COVID-19 outcomes, then the rate of COVID-19 cause-specific mortality would generally be expected to rise with the rates of obesity in a linear, consistent, dose-responsive manner.

Interestingly about 20% of all countries reported relatively high obesity rates, but fairly low rates of hypertension. These countries experienced low COVID-19 cause-specific mortality rates despite high obesity. These interactions suggest a number of areas of research on these joint behaviors and/or markers.

In Appendices B,C (supplemental information) we provide additional examples that examine policy responses and national demographics, respectively.

**Figure 6:**
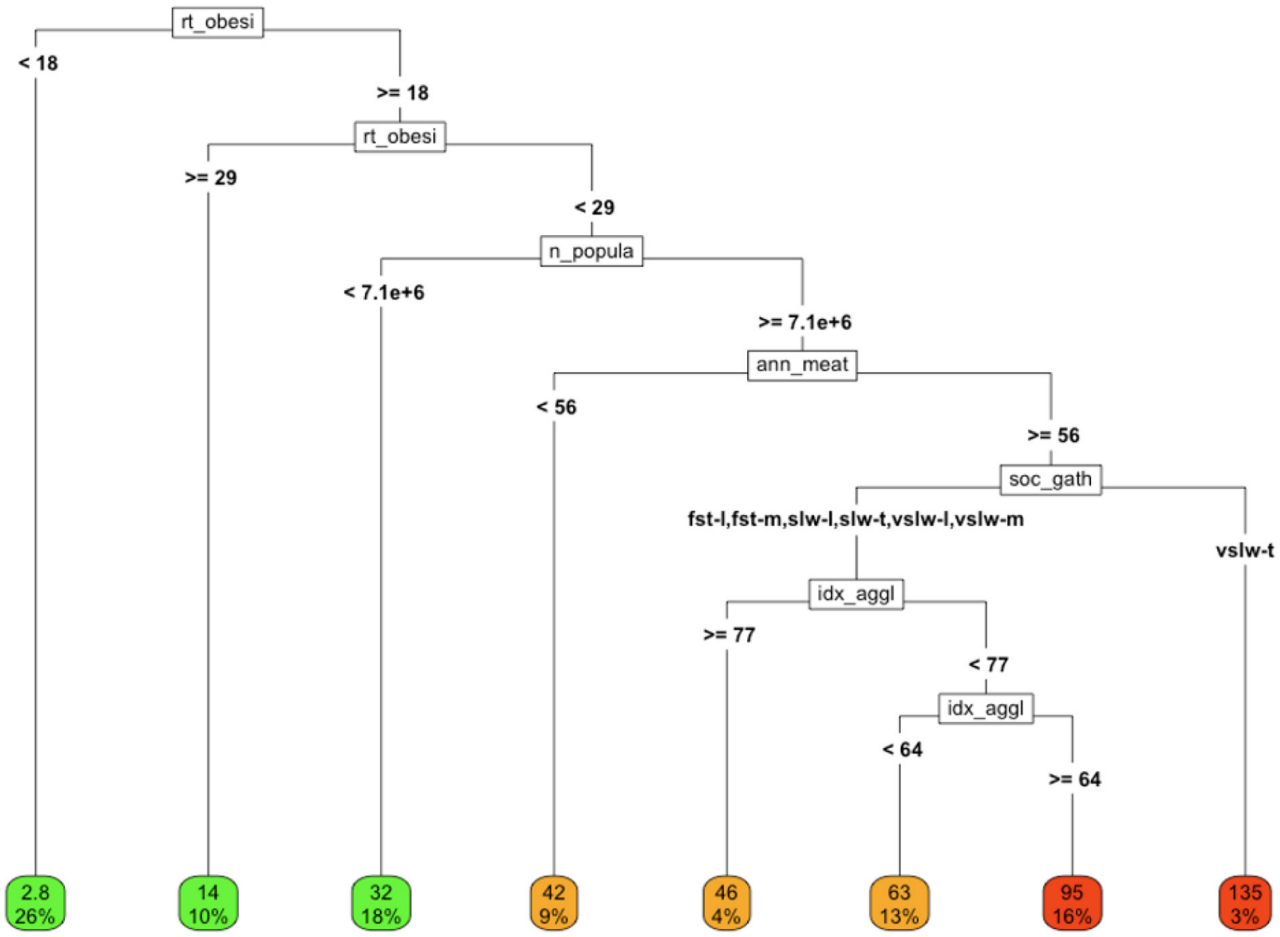
COVID-19 cause-specific mortality (deaths/100K) in association with a variety of factors from different domains. Variables in final model: rt_obesity = national obesity rate, ann_meat_consump_kg = average number of kilograms of meat consumed per capita soc_gath = social gathering policy n_population = population of country, idx_agglomeration_50K, agglomeration index (59K). Social gathering policy key; fst, fast; slw, slow; vslw, very slow; t, tight policy, m, moderately tight policy, l, loose policy; nn, no policy.

**Example 7: COVID-19 and a cross section of multidisciplinary markers**
After completing single domain analyses, we examined the impact of including a sampling of covariates from each of the domains in a single, multidisciplinary model (Figure 6).
In this analysis, *similar good outcomes* (i.e., low rates of COVID-19 cause-specific mortality per 100K) *and bad outcomes* (i.e., high rates of COVID-19 cause-specific mortality) can be observed along diverse branches of the tree, highlighting the confounding and interactions among the variables and their underlying drivers.

## Discussion

Among the salient pandemic findings to date, cardiometabolic disease and obesity have emerged as strong predictors of adverse outcomes, along with age *at an individual level*. At a *population* level, however, the influence of obesity and cardiometabolic disease appear to vary with factors in other domains, notably local economics, national demographics, public policies, and politics.

We assembled a database containing a selection of such covariates that not only spanned reporting domains, but that also spanned populations around the globe. We analyzed this data using linear and non-linear regression techniques and recursive partitioning algorithms, in order to enumerate associations both among independent covariates and COVID-19 outcomes, and among the independent covariates themselves. We found that the apparent “explanations” for COVID-19 cause-specific mortality rates across countries shifted with respect to a complex array of covariate interactions, and that simple explanations remain stubbornly elusive.

Our methods and findings suggest that a full understanding of the toll of the COVID-19 pandemic, necessary to prepare and defend better against similar future events, requires an analysis both within and among diverse covariate domains, and within and among diverse populations. Fundamentally, hypothesis testing related to pandemic outcomes must be attentive to covariate interactions, and respectful of epidemiologic context. Of note, obesity tracks with indigence in affluent countries and with affluence in indigent countries, and thus might covary with factors that increase, or decrease, vulnerability to adverse COVID outcomes (e.g., access to clinical care).

Related studies have attempted to explore potential causes that may have impacted the rates of COVID-19 cases and/or cause-specific mortality in a country, such as the association between COVID-19 deaths and prevalence of obesity in a population [e.g., (29)]. However, these studies remain mostly anecdotal and often ignore both the statistical limitations of the data and analysis, as well as other potential explanations (beyond linear or univariate relationships), that might more realistically describe the data, given the confounding of the other factors.

Our goal in compiling our dataset was not to assert a causal relationship between a specific (single) covariate and COVID-19 prevalence and cause-specific mortality. Rather, we have attempted to examine the possible interconnectedness of multiple covariates and their associations with the prevalence of COVID-19 in a country and to demonstrate why testing hypotheses about these relationships using aggregate data can be fraught. Indeed, the very presence of confounding factors often made it particularly difficult to interpret uni- and multivariate analytical results from even simple linear models (such as those estimated with OLS).

### Implications

Taken in full, our analysis has a number of implications for advancing the understanding of the COVID-19 pandemic and its aftermath, as well as for epidemiological research more broadly.

The analysis reveals that teasing out the apparent influence of even the most salient predictor covariates, especially at the population rather than individual level, can be challenging due to the influence of and interactions among many of these, which can vary dramatically with context.

Specifically, at the *population* level, the impact of obesity and cardiometabolic disease on COVID-19 cause-specific mortality rates can plausibly vary with economic development and robustness; demographics such as age distributions; the robustness and sufficiency of national infrastructure and resources; access to acute medical care; and political and policy structures and approaches.^9^

Indeed, *even the sign of the effect* of a covariate may change in different settings for structural (rather than numerical stability) reasons. While there appears to be evidence of a positive relationship at the population level between obesity rates and COVID-19 cause-specific mortality in affluent countries, the opposite appears to be true for populations that are less affluent. Thus, the answer to the question of whether obesity is associated with an increased COVID-19 cause-specific mortality rates is, “It depends.”

Importantly, the implication of this view is *not* that a reliable understanding of the pandemic is unobtainable. Rather, this analysis highlights the importance of thoughtfully structuring and integrating potential markers from a range of domains, and candidate explanations, before drawing firm conclusions about the drivers of outcomes in the COVID-19 pandemic. By construction, this requires that researchers remain skeptical of simple explanations, based on aggregate data.

### Caveats

The quality of the reported data on which we based our examples and analysis, and the protocols by which it was collected and verified varied greatly. We have deliberately demurred any discussions of the impact of data collection practices, reporting, variable definitions, coding and other variance increasing features of the data itself, and how it is transformed, though these issues are important and can critically affect inference^10^ [e.g., (30)].

We also emphasize that, consistent or not, the data underling our analyses are reported at the *population* level. Such population-level data are essential for establishing major pandemic patterns, and for evaluating population-level interventions (such as government policies and mandates).

However, in most cases it is not appropriate to generalize population-level analysis to individuals, since effects estimated at a population can be misleading when applied to individuals. (e.g., It would be wrong to conclude from Example 3 that very obese *individuals* tend to experience lower *individual* COVID cause-specific mortality and morbidity than those who are moderately obese). Such generalizations are prone to what is commonly termed the *ecological fallacy* [see, for example, (31–37)]. Ecological inference problems can arise both due to numerical issues (e.g., Jensen's Inequality), and/or due to statistical issues (e.g., Simpson's Paradox).

For concreteness, we showed examples in which, a *population* may have a high obesity prevalence and a low COVID-19 casualty toll, which may (or may not) occur because that high prevalence occurs in conjunction with individual resource repletion, a robust medical care infrastructure, etc. This can be unbundled by further partitioning.

However, ultimately, we often wish to understand how an *individual's* weight, access to capital, education, etc. affects longer-term health outcomes. This, in general, can only be understood by modeling individual-level data (e.g., within a given population with a high prevalence of, obesity, and a relatively low COVID-19 casualty toll- how did COVID-19 outcomes vary among individual population members with respect to their weight or BMI, after controlling for individual factors of seeming relevance such as education, income, insurance status, and so forth?).

## Conclusion

A full understanding of the associations among the drivers of differential COVID-19 outcomes at a national level will benefit greatly from a careful enumeration of candidate factors, an examination both within and among diverse populations, and a holistic representation of factors impacting the population of interest.

## Data availability statement

The original contributions presented in the study are included in the article/Supplementary material, further inquiries can be directed to the corresponding author.

## Author contributions

RMS: statistical methodology and analysis, drafting of content, sourcing of data, and content editing. DLK: drafting of content, review of data and analytics, sourcing of data, and content editing and revisions. All authors contributed to the article and approved the submitted version.

## Conflict of interest

The authors declare that the research was conducted in the absence of any commercial or financial relationships that could be construed as a potential conflict of interest.

## Publisher's note

All claims expressed in this article are solely those of the authors and do not necessarily represent those of their affiliated organizations, or those of the publisher, the editors and the reviewers. Any product that may be evaluated in this article, or claim that may be made by its manufacturer, is not guaranteed or endorsed by the publisher.

## Footnotes

^1^Ideally, we would like to know the fatality rate across cases. However, to do so, we would require the number of “cases” in the denominator. For to a variety of reasons that have been well documented (sub-population reporting disparities, the availability of at-home tests, etc.) this denominator is not well reported. For this reason, we have focused on cause-specific mortality, which is plausibly directionally consistent and is also likely to exhibit errors that that are orders of magnitude less severe.

^2^Note that, for reasons that will become obvious, throughout this article we use the terms “association” and “covariates” rather than causal terms such as “cause” and “drivers”.

^3^We are grateful to Natalie Nordenfelt for extensive support in gathering our data from disparate sources.

^4^Briefly, depending on the information we were seeking, we drew from: (7–17), and the associated datasets.

^5^See the Discussion section and references therein. For a more general pedagogical perspective, see, Hand (21).

^6^We also note, however, that while the loess fit is better for the high obesity rate cases, it does only marginally better on the 20–30% cases than did the linear model.

^7^Thus, for example, this would undermine, to some extent, an argument that: high-obesity lead to higher COVID-19 cause-specific mortality, which then lead to quicker pandemic policy response.

^8^We present the estimates of series of nested linear models in Supplementary Table 1 of supplemental information.

^9^We are expressly not making any assertions about the relationship between, e.g., national infrastructure and COVID-19 cause-specific mortality (though this is also evident in some cases). Rather our analysis suggests that these relationships themselves are mediated by covariations and interactions among many of the factors we examined.

^10^For example, in Supplementary Table 1 in the supplemental information, we carefully chose variables that were fully observed for all counties (i.e., in each regression, the set of countries was the same). However, had we elected to include several other of our preferred covariates, we could easily have produced regressions that were non-conformable since some of the covariates were not reported for all 68 countries. Had we instead included only those countries for which all covariates were observed, we would have made the regressions conformable, but might have systematically excluded those countries that did not have the resource or infrastructure to collect this data or which did not collect it for some other reason.
