# Supplementary material for: Interpreting global variations in the toll of COVID-19: The case for context and nuance in hypothesis generation and testing
Source: Front Public Health. 2022 Oct 19;10:1010011. doi: 10.3389/fpubh.2022.1010011 (PMC9627160; doi:10.3389/fpubh.2022.1010011)
Supplement: Supplementary file 1 [file Data_Sheet_1.docx]

# *Supplemental Information for*

# Global Variations in the Toll of COVID19:

# The Case for Context and Nuance in

# Hypothesis Generation & Testing

# NOTE TO EDITOR: ALL OR SOME OF THESE APPENDICES MAY BE EITHER MOVED AFTER THE REFERENCES IN THE MAIN PAPER OR HOSTED ONLINE AS SUPPLEMENTAL INFORMATION

# Appendix A: Univariate and nested linear models incorporating factors from multiple domains

|  | **Model 1** | **Model 2** | **Model 3** | **Model 4** | **Model 1-2** | **Model 1-3** | **Model 1-4** | **Model 1-234** |
| --- | --- | --- | --- | --- | --- | --- | --- | --- |
| Constant | 2.24 | -0.33 | -39.10 | 69.77** | -12.31 | -47.95* | 28.44 | -53.49 |
|  | (11.44) | (17.32) | (24.97) | (22.49) | (16.94) | (23.85) | (23.44) | (46.89) |
| Obesity Rate | 1.95*** |  |  |  | 1.67** | 1.56** | 1.93*** | 1.43* |
|  | (0.52) |  |  |  | (0.58) | (0.53) | (0.52) | (0.58) |
| Agglomeration Idx (50K) |  | 0.68* |  |  | 0.33 |  |  | 0.17 |
|  |  | (0.27) |  |  | (0.28) |  |  | (0.29) |
| Median Life Exp. |  |  | 174.48** |  |  | 126.08* |  | 118.70+ |
|  |  |  | (53.24) |  |  | (53.09) |  | (68.08) |
| Income inequality (10%/50%) |  |  |  |  |  |  | -10.05 | 0.29 |
|  |  |  |  |  |  |  | (7.87) | (9.52) |
| Num.Obs. | 68 | 68 | 68 | 68 | 68 | 68 | 68 | 68 |
| *R^2^* | 0.17 | 0.09 | 0.14 | 0.03 | 0.19 | 0.24 | 0.19 | 0.24 |
| *F* | 13.9 | 6.9 | 10.7 | 1.7 | 7.7 | 10.3 | 7.8 | 5.1 |
| *+ p < 0.1, * p < 0.05, ** p < 0.01, *** p < 0.001* | | | | | | | | |

**Variance Inflation Factors Model 1-234**

| rt_obesity | 1.29 |
| --- | --- |
| idx_agglomeration_50K | 1.31 |
| rt_med_life_yrs | 1.78 |
| log(inc10vs50) | 1.52 |

Table 1 Nested models of disparate factors and variance inflation factors for full model

# Appendix B: COVID-19 Mortality and various policy factors

We examined policy responses as markers of risk. These are shown in the figure below. In this case, the picture is even murkier: similar mortality ranges were observed across very diverse responses- fast versus slow, restrictive versus loose-, etc., likely because the similar policies were implemented in different contexts.

For example, our data appear to suggest that, in terms of COVID-19 outcomes, there is clustering of social distancing policies that, on the surface, seem quite different (note labeling on left most two branches). Those policies that were implemented quickly, but which were not very restrictive as well as those policies that were implemented more slowly, but which were stricter- were statistically similar with respect to COVID outcomes.


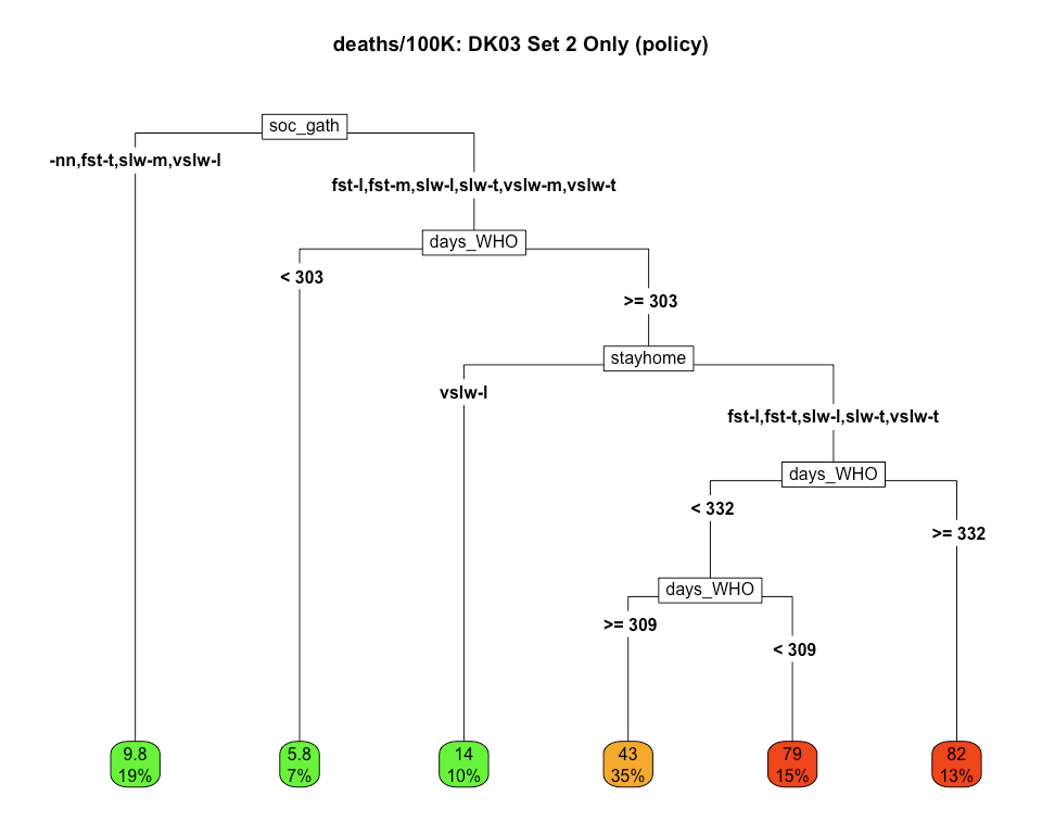


Figure 7 COVID-19 mortality (deaths / 100K) in association with policy responses. Key: fst = fast, slw = slow, vslw = very slow; t = tight policy, m = moderately tight policy, l = loose policy; nn = no policy. Variables in final model: soc_gath = social gathering policy, days_WHO_report2testing = # of days between WHO classification of COVID-19 outbreak as a pandemic and commencement of testing policy stayhome = stay home policy.

However, within these clusters, the COVID-19 outcomes varied greatly depending on other factors not readily evinced by the model. For instance, a slow and loose policy response might be the result of (a) disagreements regarding politics, economic concerns and ideology, despite a high case count, or (b) a low case count and lower perceived urgency. The diversity of the design and implementation of various global responses undoubtedly affects both of these.

# Appendix C: COVID-19 mortality and various demographic factors

We examined demographic covariates, the resulting tree-model for which is shown in the figure below. Here, *low rates of mortality* were observed *both* in countries with relatively l*ow GDP per capita* and with *higher GDP per capita* provided that *income inequality* was also relatively high.

The *highest mortality rates* were observed in instances in which a country was:

- densely highly urbanized (Agglomeration Index), but in which
- there is evidence of economic hardship, as measured by relatively high unemployment (rightmost node).

In isolation, this analysis would omit both obesity and policy in explaining adverse COVID-19 outcomes, and we might instead propose the importance of urban living, crowding, and economic hardship/unemployment.


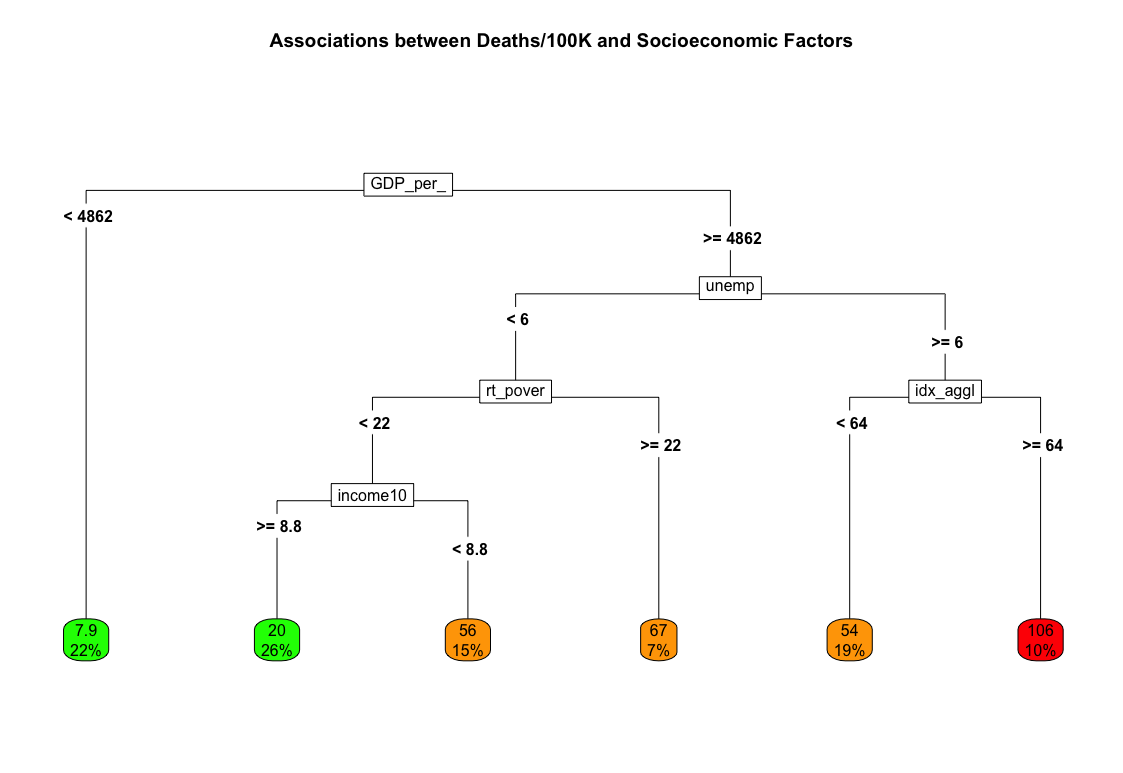


Figure 8 COVID-19 mortality (deaths / 100K) in association with socio-economic factors. Variables in final model: GDP_per_capita = per capita GDP, unemp = national unemployment rate, rt_poverty = percentage of population at or below the poverty line; idx_agglomeration_50K = agglomeration index (59K), income_10vs50 = ratio of the top 10^th^ percentile income to the median income,

Countries with *higher GDP per capita*, *lower unemployment* and l*ower poverty rates* fared better than those countries with similar GDP per capita, but which also had either higher unemployment or higher poverty rates. Indeed, the second-best performing outcomes were in the cluster that included countries with high GDP per capita, *lower* poverty but *higher* income inequality, while the worst performing cluster included high GDP countries with higher unemployment, *particularly when these countries were more urbanized.*

There are a variety of hypotheses that might explain these results. For example, it could be the case that high GDP per capita, lower poverty and *higher* income inequality countries are those in which the general level of income is relatively favorable, but in which there may also be a fair number of extremely affluent members of the population, but in which a substantial proportion of lower income individuals are still above the poverty line. However, it is equally plausible, that there is not an exploitable pattern in our data with respect to examining *only* economic indicators in isolation.

# Appendix D: Data sources and conventions

Data on COVID-19 positivity and mortality rates were collected from the World Health Organization COVID-19 Dashboard (WHO, 2020). These data included, for each country: ^[[1]](#footnote-1)^

- the date of first reported COVID-19 case in the country;
- the total number of COVID-19 cases and deaths;
- the peak number of new COVID-19 cases; and
- the date of the peak.

We used these raw reported data to compute various derived variables from these statistics, to make all measures roughly conformable (e.g., COVID-19 cases and deaths per 100K residents, etc.).

We collected several *health statistics* at the population level:

- obesity rate (Our World in Data, 2020);^[[2]](#footnote-2)^
- hypertension rate (WHO, 2020); and
- total volume of meat consumed per capita (Our World in Data, 2020).^[[3]](#footnote-3)^

The statistics on *national economic indicators* included:

- unemployment rate (UN, 2020a);^[[4]](#footnote-4)^
- median household income; ^[[5]](#footnote-5)^
- GDP and GDP per capita (Worldometer, 2020);^[[6]](#footnote-6)^
- poverty rate/ percent of population below the poverty line (CIA, 2020); ^[[7]](#footnote-7)^ and
- measures of income inequality (Chancel, *et al.*, 2021).

The statistics on each country’s *demographic features* (CIA, 2020, unless noted otherwise) included:

- population count;
- geographic size (area in km^2^);
- median age;
- average life expectancy;
- population age distribution;^[[8]](#footnote-8)^ and
- the Agglomeration Index, a composite measure of each country’s urban concentration (Nelson and Uchida, 2010).^[[9]](#footnote-9)^

Data on each country’s *health infrastructure and international connectedness* included:

- the number of hospital beds per 1000 inhabitants (World Bank, 2020);^[[10]](#footnote-10)^
- the number of yearly international tourist departures (UNWTO, 2020a);^[[11]](#footnote-11)^
- the number of international visitors (UNWTO, 2020b);^[[12]](#footnote-12)^ and
- the Henley Passport Index, a measure of “passport power” (H&P, 2020); ^[[13]](#footnote-13)^
- Data on each country’s COVID-19 policy interventions included descriptions of a country’s policies and mandates relating to (Hale, *et al.*, 2020 and underlying data);^[[14]](#footnote-14)^
- COVID-19 testing;
- contact tracing;
- travel restrictions;
- social gathering restrictions;
- workplace closure mandates;
- school closure mandates; and
- shelter in place/ stay at home orders.

We coded these free-text data with respect to policy classes (e.g., COVID testing, workplace closure, etc.). We then recorded the start date of each policy measure in each country. We also coded the stringency of the mandate or policy. Finally, we created a derived classification of each policy in each country based on combining these two classes (e.g., “fast-tight”, “slow-loose”, etc.)

1. Reported as of 28 December, 2020. [↑](#footnote-ref-1)
2. Obesity rate is defined as the percentage of a population with a BMI > 30.0. [↑](#footnote-ref-2)
3. Per capita meat consumption is reported in kg, as of 2017. [↑](#footnote-ref-3)
4. Unemployment rates per country for year 2019. [↑](#footnote-ref-4)
5. Median household income was reported in $US. [↑](#footnote-ref-5)
6. Reported as of 2017. [↑](#footnote-ref-6)
7. Reported for various years. Most recent year used. [↑](#footnote-ref-7)
8. Age distribution estimates for 2020 (in thousands) were binned into age brackets: 0-14, 15-24, 25-44, 45-54, 55-64, 65-84, and 85+ years. [↑](#footnote-ref-8)
9. The Agglomeration Index (AI) is intended as a globally consistent metric of urbanization. The AI incorporates three factors: population density, population size (count), and the time it takes to travel to the center of a given urban center from various points in the country. The AI is calculated by first one defining criteria for (a) population size and (b) population density that constitute a “large” urban area; and (c) the maximum travel times to the center of the urban center, using the fastest mode of transportation available such that the urban center is still considered “accessible” from the location, The AI is then computed as: the total number of individuals living in areas that meet all three criteria, divided by the total population of the country. [↑](#footnote-ref-9)
10. Reported for various years. Most recent year used. (*See:* <https://data.worldbank.org/indicator/SH.MED.BEDS.ZS>) [↑](#footnote-ref-10)
11. Reported for the years 2017 and 2018. Most recent year used. [↑](#footnote-ref-11)
12. For variable years. [↑](#footnote-ref-12)
13. Data for 2020 used. The Henley Passport Index is calculated from International Air Transport Association (IATA) data and analysis by Henley & Partners. Each nation’s passport is scored on the total number of destinations that the passport holder can access without a visa (*see*: <https://www.henleyglobal.com/passport-index> ). [↑](#footnote-ref-13)
14. *See*: <https://www.bsg.ox.ac.uk/research/publications/variation-government-responses-covid-19> . [↑](#footnote-ref-14)
